# Achieving universal health coverage

**DOI:** 10.2471/BLT.14.149070

**Published:** 2015-08-14

**Authors:** Joseph Wong

**Affiliations:** a Political Science and Munk School of Global Affairs, University of Toronto, 1 Devonshire Place, Toronto, Ontario M5S 3K7, Canada.

There is global consensus on the goals of universal health coverage (UHC), which has been defined as “all people receiving quality health services that meet their needs without exposing them to financial hardship in paying for them.”^1^ Yet despite this consensus, it remains unclear how the global health community can achieve universal coverage. The obvious answer is to ensure that health services reach those who need them. The complicated real-world challenge, however, is actually delivering services to reach those most in need and more specifically to those who are hardest to reach. Without a focus on reach, resources will be wasted and the number of preventable deaths and treatable illnesses will remain high.

Until now, the main constraint in achieving UHC has been understood in terms of lack of ability to pay for health services. The barrier to universal health, many argue, is the un-affordability of health services for the world’s very poor.^1^ Hence, conventional wisdom prescribes the reduction of financial barriers – such as the removal of user fees – to achieve UHC.^2^ This is not the wrong prescription; it is an important one and without a reduction of such financial barriers, UHC is impossible. But it is a prescription that only addresses one obstacle.

As the global health community moves beyond the financial model of health access, it has begun to consider and address additional barriers to health care, such as geographical distance, cultural differences, gender norms, citizenship, social determinants and so on.^1^ Innovative methods are needed so that health services reach beyond and around these barriers. After all, a very poor villager in the rural hinterland of India is more likely to be unhealthy not just because she cannot afford health care, but also because many health services do not reach her.

But to be reached requires one to be visible in the first place. If the goal of UHC is to reach vulnerable populations who are otherwise left out, then all people need to be visible. Many people in high-income countries take visibility for granted. Most pay taxes; have an official identification; a registered birth; a street address. They can be reached by health services because they are visible.

This is not the reality for many people in low- and middle-income countries, particularly those who are very poor or marginalized. For instance, how is it possible to deliver an immunization programme in a slum where no one has an address and thousands are constantly on the move? Anthropologist Arjun Appadurai, in his work on slums in Mumbai, laments that in India “a host of local, state-level and federal entities exist with a mandate to rehabilitate or ameliorate slum life. But none of them knows exactly who the slum dwellers are, where they live, or how they are to be identified.”^3^

The problem of invisibility, especially among the world’s poor, is not easily overcome. Most poor workers in low- and middle-income countries are employed informally, irregularly or casually and as a result are fiscally invisible;^4^ hundreds of millions live in slums – illegal settlements without official addresses;^5^ stateless peoples such as the Roma in Europe and the “hill peoples” in Asia are excluded from the health care benefits of citizenship.^6^ Until recently, nearly half of India’s population had no formal identification; in China, the household registration system means that hundreds of millions of migrant workers are ineligible for social benefits;^7^ in Israel, unrecognized Bedouin villages are literally off the official map. According to the United Nations Children’s Fund (UNICEF), an estimated 230 million births are unregistered worldwide, most of whom are born into poor households and are therefore likely to be excluded from health services.^8^

To address the problem of reach requires methods that actively connect the provision of health services to people who are otherwise invisible and thus unreachable. There are successful examples, including Brazil’s conditional cash transfer programme – *Bolsa Família* – implemented in 2003. Close to 14 million families identified as being in the bottom income quintile have been enrolled in the program. *Bolsa Família* provides a direct cash transfer to poor families, on the condition that their children attend school, are immunized and have regular medical check-ups. In tandem with Brazil’s *Programa Saúde da Família*, these conditional cash transfers have had a positive effect on child health, including a significant decrease in under-5 mortality rates.^9^ The programme has been very successful in reaching poor families, with around 75% of cash transfers reaching the intended beneficiaries. (In other Latin American countries, similar programs transfer only 35–50% of resources to the intended people).^10^ The programme has had a positive health impact among the poor because it has effectively reached the poor.

There are several reasons why the *Bolsa Família* programme has been so successful in extending its reach. First, it is a targeted programme, explicitly mandated to reach the very poor. Second, it is very precise in its targeting. A Brazilian official once remarked that “if all the resources spent on social policies in Brazil were dropped from a helicopter, they would have a better chance of reaching the poor than they have now.”^11^ The *Bolsa Família* programme addresses such imprecision by implementing a sophisticated census exercise, constructing poverty maps and a unified registry and employing technical solutions for monitoring within municipalities. The programme was helped by the existing work card system, near universal birth registration rates and a mandatory voting law, meaning all voting-age citizens are registered. Third, the program, while funded by the federal government, is administered locally, along with the *Programa Saúde da Família*, in over 5000 municipalities. The administration is closer to the ground, where local agents possess better local knowledge. Local administration apparently reduces corruption – a frequent problem in the delivery of social services – and allows for more flexible implementation.^10^

Reaching the unreached is expensive, as the World Health Organization has recently acknowledged.^1^ The *Bolsa Família* programme relies on costly interventions, including establishing and maintaining the unified registry; implementing the card technology used to transfer and access funds; installing cashpoint machines, especially in difficult-to-reach places; providing incentives to continue to identify inclusion and exclusion errors in enrolment and expending resources to ensure that the required conditions (school attendance and medical care) are being met. The political and economic tension is to reconcile expanding coverage of existing health services with ensuring that services are targeted to the hardest-to-reach people. When it comes to precise targeting, there are few economies of scale to be realized, as the marginal cost of reaching each additional person or family increases for those that are hardest-to-reach.

Extending reach, therefore, is not just a technical problem to be solved but also a political problem of resource redistribution. First, stakeholders in government, nongovernmental organization and health providers should use empirical methods to evaluate the marginal cost of extending reach. This will help decision-makers devise ways to mitigate some of the costs, improve understanding of what services to include and where efficiencies can be gained. Second, programme planners and politicians must highlight the benefits – such as economic productivity and poverty reduction – of costly but ultimately effective policies. Third, because reach involves designing means and methods that connect people to health services, local innovation and adaptation are critical.

The success of the *Bolsa Família* programme can be attributed in part to the tremendous flexibility and innovation capacity that municipalities have to reach families in their jurisdictions. Local innovation is not about big fixes, but small and effective adaptations.^12^ Finally, greater investment in information and identification systems is needed. The unified registry in Brazil, the unique identification programme in India, birth registration in South Africa – if implemented effectively, can make poor and vulnerable people more visible. Having such infrastructure in place means the state and other providers can deliver other important services. The allocation of national development resources and international development assistance should support health service delivery to people who are hard to reach.
